# Special Issue “Host Targeted Therapeutics Against Virus Infections”

**DOI:** 10.3390/v16121825

**Published:** 2024-11-25

**Authors:** Stephan Pleschka

**Affiliations:** 1 Institute of Medical Virology, Justus Liebig University Giessen, 35392 Giessen, Germany; stephan.pleschka@viro.med.uni-giessen.de; 2 German Center for Infection Research (DZIF), Partner Site Giessen, 35392 Giessen, Germany

## 1. Introduction

The COVID-19 pandemic, along with the emergence and sustained transmission of highly pathogenic avian influenza viruses (H5N1) in U.S. dairy cattle, as well as the recurrent appearance of other viruses with high public health impact, underscores the critical need for effective antiviral treatments and therapies. Although vaccines remain the cornerstone of preventive strategies when available, targeted treatments for infected individuals are frequently lacking. Given that viral replication depends on host cellular machinery, there is growing interest in host-directed antiviral strategies, known as host-targeted antivirals (HTAs). HTAs hold the potential not only to combat a single virus strain but also to exhibit broad-spectrum antiviral activity across multiple members within and even beyond a virus family. This host-targeted approach is less prone to resistance development, as it interferes with host cellular factors rather than viral components encoded by the virus itself. Moreover, HTAs can be pre-manufactured or rapidly deployed in response to outbreaks. Once controversial, the concept of targeting host cellular functions for antiviral therapy has gained substantial acceptance over the past two decades. The five research articles and two reviews featured in this Special Issue present novel findings in the realm of host-targeted antiviral strategies and therapeutic interventions.

## 2. Contributions

In the first publication of this Special Issue, Jonatan C. S. de Carvalho et al. [1] (and citations therein) provide evidence that glucocorticoids (GCs), known primarily for their role in suppressing pro-inflammatory cytokines and lipid mediators, may offer protective pharmacological benefits against COVID-19-related inflammation. The study suggests that GCs could reduce levels of platelet-activating factor (PAF), a key lipid mediator implicated in COVID-19 pathogenesis, while simultaneously increasing lipid mediators from the endocannabinoid (eCB) group, which play a crucial role in modulating an effective innate immune response during inflammation. Based on these findings, the authors propose potential therapeutic strategies targeting different phases of lipid mediator metabolism to modulate immune responses for COVID-19 treatment.

Charlotte Foret-Lucas et al. [2] (and citations therein) demonstrate that inhibiting EPAC1 with the specific pharmacological agent AM-001 effectively suppresses early stages of the SARS-CoV-2 replication cycle. The authors report a concentration-dependent reduction in the release of infectious SARS-CoV-2 and H1N1 influenza A virus particles (measured by TCID50 assay), as well as a decrease in SARS-CoV-2 viral RNA release (quantified by RT-qPCR). Notably, AM-001 treatment showed no virucidal activity and did not significantly affect cellular viability, indicating a targeted mechanism of action. These findings suggest that EPAC1 inhibition may serve as a promising therapeutic strategy against selected viral infections.

In their study, Tosin Oladipo Afowowe et al. [3] (and citations therein) identified topoisomerase II as a novel antiviral target for panarenaviral diseases. Utilizing modified minigenome (MG) systems for Lassa virus (LASV) and Junin virus (JUNV), which cause Lassa fever and hemorrhagic fever with mortality rates of 15–30% and up to 30% [4], respectively, the authors screened both an FDA-approved compound library and additional agents. They found that topoisomerase II inhibitors suppressed LASV and JUNV MG activity and significantly limited JUNV infection. Additionally, siRNA knockdown of topoisomerase IIα and IIβ reduced JUNV replication, suggesting that topoisomerase II could serve as a molecular target for broad-spectrum panarenaviral inhibitors.

Wiebke Obermann et al. [5] (and citations therein) investigated rocaglates, such as silvestrol, as antiviral agents. These plant-derived compounds inhibit viral protein synthesis across various RNA viruses by clamping 5′-UTRs of viral mRNAs to the RNA helicase eIF4A, thus blocking initiation of translation [6]. Using reporter systems with 5′-UTRs from human coronavirus 229E (HCoV-229E), MERS-CoV, and SARS-CoV-2, the authors found that silvestrol, along with synthetic rocaglates CR-1-31-B and zotatifin, exerted dose-dependent antiviral effects, with distinct profiles in cytotoxicity and efficacy. Notably, HCoV-229E showed no resistance to rocaglates under serial passaging, highlighting their potential as broad-spectrum antivirals effective at nanomolar concentrations.

Beatriz Sierra et al. [7] (and citations therein) utilized multi-tissue transcriptomics for in silico-guided drug selection, followed by functional assessment against Dengue fever. By analyzing transcriptomic data from liver, spleen, and blood samples of both infected and non-infected deceased individuals, the study identified common host mechanisms that could be targeted by known compounds to mitigate severe Dengue virus infection. The authors propose that these identified compounds represent promising candidates for further functional evaluation and clinical trials.

In their review, Jens Kleinehr et al. [8] examine metabolic alterations induced by common respiratory viruses and explore their potential as novel antiviral targets. Given their reliance on host cells, respiratory viruses—responsible for frequent human lung infections—significantly modify cellular functions to favor viral replication, notably altering metabolic pathways. The authors provide a comprehensive summary of current knowledge on metabolic pathway modifications driven by acute respiratory syncytial virus, rhinovirus, influenza virus, parainfluenza virus, coronavirus, and adenovirus, identifying potential therapeutic targets and compounds for antiviral strategies.

In their review, Ralf Kircheis et al. [9] examine the disparity in COVID-19 mortality between heavily impacted regions such as Europe and the Americas and regions such as Africa, which reported lower infection and death rates by 2021. Analyzing this “African paradox”, the authors explore correlations among immunological and genetic factors, pre-existing immune status, cytokine induction patterns, and epidemiological factors. They propose that a young population with lower comorbidity rates, high prevalence of helminth and malaria infections, and widespread use of anti-helminth and anti-malaria drugs with anti-inflammatory properties may contribute to the observed outcomes, emphasizing the role of the NF-κB pathway. The authors suggest that understanding these epidemiological and immunological correlations could guide the development of COVID-19 therapies, including the repurposing of existing NF-κB inhibitors.

## 3. Conclusions and Outlook

This Special Issue showcases diverse antiviral strategies targeting host factors and mechanisms, underscoring the significant therapeutic potential of HTAs in combating a range of viral threats. Key approaches include the modulation of pro-inflammatory factors and pathways, disruption of cellular factors and mechanisms essential for viral gene expression and genome replication, and targeting of metabolic pathways crucial for specific viruses. Collectively, these strategies illustrate an expanding array of potential tactics that, with advancing methodologies, can be further refined to offer effective options for improved viral disease control in the future.
